# Preparation of Protein Lysates Using Biorthogonal Chemical Reporters for Click Reaction and in-Gel Fluorescence Analysis

**DOI:** 10.21769/BioProtoc.5114

**Published:** 2024-11-20

**Authors:** Yaxin Xu, Tao Peng

**Affiliations:** State Key Laboratory of Chemical Oncogenomics, School of Chemical Biology and Biotechnology, Peking University Shenzhen Graduate School, Shenzhen, China

**Keywords:** Click chemistry, Bioorthogonal chemical reporter, Metabolic labeling, In-gel fluorescence, Post-translational modifications

## Abstract

Bioorthogonal chemical reporters are non-native chemical handles introduced into biomolecules of living systems, typically through metabolic or protein engineering. These functionalities can undergo bioorthogonal reactions, such as copper-catalyzed alkyne-azide cycloaddition (CuAAC), with small-molecule probes, enabling the tagging and visualization of biomolecules. This approach has greatly enhanced our understanding of cellular dynamics, enzyme targeting, and protein post-translational modifications. Herein, we report a protocol for preparing protein lysates for click reaction and in-gel fluorescence analysis, exemplified using alk-16, a terminal alkyne-functionalized stearic acid analog, to investigate proteins with fatty acylation. This protocol provides methods for the fluorescent visualization of chemical reporter–labeled proteomes or individual proteins of interest (POIs).

Key features

• Metabolic incorporation of bioorthogonal chemical reporters into proteins in living cells.

• Visualization of proteomes or specific proteins labeled with chemical reporters via in-gel fluorescence analysis.

• Reliable, non-radioactive methods for investigating protein fatty acylation and other post-translational modifications.

## Background

**Figure 1.**
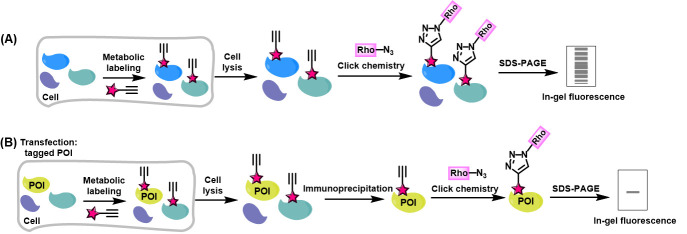
Workflow for in-gel fluorescence analysis of alkynyl chemical reporter–labeled proteins. (A) Schematic for in-gel fluorescence analysis of chemical reporter-labeled proteomes. (B) Schematic for in-gel fluorescence analysis of chemical reporter-labeled protein of interest (POI).

Bioorthogonal chemical reporters refer to non-native, non-perturbing chemical handles that can be specifically modified in living systems through highly selective reactions with exogenously delivered probes [1]. The bioorthogonal chemical reporter strategy involves incorporating unique functionalities, such as azide, alkyne, or alkene groups, into target biomolecules using the cellular biosynthetic machinery. Chemical labeling with small-molecule probes is then achieved via bioorthogonal click reactions, which are characterized by exceptional biorthogonality, biocompatibility, rapid kinetics, and high specificity in biological environments [1]. A prominent example of such click reactions is the copper-catalyzed alkyne-azide cycloaddition (CuAAC). In this reaction, an alkyne-tagged molecule reacts with an azide-tagged molecule in the presence of copper (I) to form a stable 1,4-disubstituted 1,2,3-triazole product via a [3+2] cycloaddition [2,3]. Over the past two decades, these strategies have enabled precise tracking and analysis of proteins and other biomolecules in complex biological environments, revolutionizing our understanding of biological systems. They have been applied to monitoring dynamic changes in cellular activity, profiling various cell types, states, or mutations, identifying enzyme targets, and exploring a wide range of post-translational modifications (PTMs) [4]. Herein, we present a detailed experimental protocol for the preparation of protein lysates for click reaction and in-gel fluorescence analysis, using alk-16 as a representative example. Alk-16 is a terminal alkyne-functionalized stearic acid analog that mimics endogenous long-chain fatty acids and can undergo a click reaction, allowing for the investigation of proteins with fatty acylation [5–7]. This includes the metabolic incorporation of chemical reporters into proteins in living cells, global visualization of reporter-labeled proteins in gels by selectively reacting alkynyl chemical reporter–labeled proteins in cell lysates with azido-rhodamine, and the analysis of reporter-labeled candidate proteins using immunoprecipitation, click chemistry, and fluorescence scanning (Figure 1). We aim to provide a general protocol for in-gel fluorescence analysis of proteins that are labeled by bioorthogonal chemical reporters. While we use alk-16 as an example to describe the protocol, it is also adaptable to other bioorthogonal chemical reporters, such as alkynyl-functionalized YnLac [8] and HMGAMyne [9] to detect and identify the cellular lactylated and HMGylated proteins.

## Materials and reagents

**Reagents**

1. DMSO (Sigma-Aldrich, catalog number: D2650)
2. DMEM (Thermo Fisher Scientific, catalog number: 11995065) or other culture media for growing the cell type of interest
3. Fetal bovine serum (FBS) (Thermo Fisher Scientific, catalog number: 26140079)
4. Charcoal/dextran-treated fetal bovine serum (Cytiva, catalog number: SH30068)
5. Opti-MEM (Thermo Fisher Scientific, catalog number: 31985070)
6. ViaFect transfection reagent (Promega, catalog number: E4981)
7. Benzonase (Sigma-Aldrich, catalog number: E1014)
8. EDTA-free protease inhibitor cocktail (Roche, catalog number: 11873580001)
9. Sodium chloride (NaCl) (Sigma-Aldrich, catalog number: S3014)
10. Potassium chloride (KCl) (Sigma-Aldrich, catalog number: P9541)
11. Sodium phosphate dibasic (Na_2_HPO_4)_ (Sigma-Aldrich, catalog number: S3264)
12. Potassium phosphate monobasic (KH_2_PO_4_) (Sigma-Aldrich, catalog number: P9791)
13. Sodium dodecyl sulfate (SDS) (Sigma-Aldrich, catalog number: L3771)
14. Trizma hydrochloride (Tris-HCl) (Sigma-Aldrich, catalog number: T3253)
15. Hydrochloric acid (HCl) (Sigma-Aldrich, catalog number: 258148)
16. Triton X-100 (Sigma-Aldrich, catalog number: T8787)
17. Sodium deoxycholate (Sigma-Aldrich, catalog number: D6750)
18. Triethanolamine (TEA) (Sigma-Aldrich, catalog number: 90279)
19. 4-(2-Hydroxyethyl)-1-piperazineethanesulfonic acid (HEPES) (Sigma-Aldrich, catalog number: 54457)
20. BCA assay reagents (Thermo Fisher Scientific, catalog number: A55864)
21. Red anti-HA affinity gel (Sigma-Aldrich, catalog number: E6779)
22. Red anti-FLAG M2 affinity gel (Sigma-Aldrich, catalog number: F2426)
23. Anti-GFP nanobody agarose beads (AlpaLifeBio, catalog number: KTSM1301)
24. Tert-butanol (t-BuOH) (Sigma-Aldrich, catalog number: 471712)
25. Methanol (Sigma-Aldrich, catalog number: 34860)
26. Glycerol (Sigma-Aldrich, catalog number: G5516)
27. Bromophenol blue (Sigma-Aldrich, catalog number: B8026)
28. Bond-breaker TCEP solution, neutral pH (Thermo Fisher Scientific, catalog number: 77720)
29. Hydroxylamine hydrochloride (NH_2_OH·HCl) (Sigma-Aldrich, catalog number: 55460)
30. Tween-20 (Sigma-Aldrich, catalog number: 655205)
31. Acetic acid (Sigma-Aldrich, catalog number: A6283)
32. Clarity western ECL substrate (Bio-Rad, catalog number: 1705060)
33. Alk-16 (Sigma-Aldrich, catalog number: O8382; alternatively, it can be synthesized as described in Charron et al. [5])
34. Azido-rhodamine (the synthetic method has been described in Charron et al. [5])
35. Tris(2-carboxyethyl) phosphine hydrochloride (TCEP) (Sigma-Aldrich, catalog number: C4706)
36. Tris[(1-benzyl-1H-1,2,3-triazol-4-yl)methyl]amine (TBTA) (Sigma-Aldrich, catalog number: 678937)
37. Copper sulfate pentahydrate (CuSO_4_·5H_2_O) (Sigma-Aldrich, catalog number: C8027)
38. Tris-MOPS-SDS running buffer (GenScript, catalog number: M00138)
39. Coomassie Brilliant Blue staining solution (Beyotime, catalog number: P0003S)

**Solutions**

1. 50 mM alk-16 (see Recipes)
2. PBS (1 L) (see Recipes)
3. RIPA buffer (see Recipes)
4. 1% SDS lysis buffer (see Recipes)
5. 4% SDS buffer (see Recipes)
6. HEPES buffer (see Recipes)
7. 2 M NH_2_OH solution (see Recipes)
8. 5 mM azido-rhodamine stock solution (see Recipes)
9. 50 mM TCEP solution (see Recipes)
10. 2 mM TBTA stock solution (see Recipes)
11. 50 mM CuSO_4_ solution (see Recipes)
12. 4× SDS-PAGE loading buffer (see Recipes)
13. Destaining buffer (see Recipes)

**Recipes**

1. **50 mM alk-16**
  Dissolve 14.0 mg of alk-16 in 1 mL of DMSO.
2. **PBS (1 L)**
  - Measure and mix the following components:
    8 g of NaCl (137 mM)
    0.2 g of KCl (2.7 mM)
    1.44 g of NaHPO (10 mM)
    0.24 g of KHPO (1.8 mM)
  - Add ultrapure water to a final volume of 1 L.
  - Adjust the pH to 7.3 using HCl.
3. **RIPA buffer (1 L)**
  - Measure and mix the following components:
    10 mL of Triton X-100 (1% v/v)
    10 g of sodium deoxycholate (1% w/v)
    1 g of SDS (0.1% w/v)
    8.78 g of NaCl (150 mM)
    50 mL of 1 M Tris-HCl pH 7.4 (50 mM)
  - Add ultrapure water to a final volume of 1 L.
4. **1% SDS lysis buffer (1 L)**
  - Measure and mix the following components:
    10 g of SDS (1% w/v)
    8.78 g of NaCl (150 mM)
    50 mL of 1 M HEPES pH 7.4 (50 mM)
  - Add ultrapure water to a final volume of 1 L.
5. **4% SDS buffer (1 L)**
  - Measure and mix the following components:
    40 g of SDS (4% w/v)
    8.78 g of NaCl (150 mM)
    50 mL of 1 M TEA pH 7.4 (50 mM)
  - Add ultrapure water to a final volume of 1 L.
6. **HEPES buffer (1 L)**
  - Measure and mix the following components:
    8.78 g of NaCl (150 mM)
    50 mL of 1 M HEPES pH 7.4 (50 mM)
  - Add ultrapure water to a final volume of 1 L.
7. **2 M NH_2_OH solution (10 mL)**
  - Dissolve 1.39 g of NH_2_OH·HCl in 8 mL of ultrapure water.
  - Adjust the pH to 7.4 using NaOH.
  - Add ultrapure water to a final volume of 10 mL.
8. **5 mM azido-rhodamine stock solution**
  Dissolve 34.3 mg of azido-rhodamine in 10 mL of DMSO.
9. **50 mM TCEP solution**
  Dissolve 14.3 mg of TCEP in 1 mL of ultrapure water.
10. **2 mM TBTA stock solution**
  Dissolve 10.6 mg of TBTA in 10 mL of a 1:4 (v/v) mixture of DMSO and t-BuOH.
11. **50 mM CuSO_4_·5H_2_O solution**
  Dissolve 12.5 mg of CuSO_4_·5H_2_O in 1 mL of ultrapure water.
12. **4× SDS-PAGE loading buffer (100 mL)**
  - Measure and mix the following components:
    8 g of SDS (8% w/v)
    20 mL of 1 M Tris-HCl pH 6.8 (200 mM)
    40 mL of glycerol (40% v/v)
    0.4 g of bromophenol blue (0.4% w/v)
  - Add ultrapure water to a final volume of 100 mL.
13. **Destaining buffer (1 L)**
  - Measure and mix the following components:
    500 mL of methanol (50% v/v)
    100 mL of acetic acid (10% v/v)
    400 mL of ultrapure water (40% v/v)
  - Mix thoroughly to ensure the components are well combined.

**Laboratory supplies**

1. 60 mm TC-treated culture dish (Corning, catalog number: 430166)
2. 6-well clear TC-treated multiple-well plate (Corning, catalog number: 3516)
3. Pipette tips (Rainin, catalog numbers: 30180889, 30374583, 30296781)
4. 10 mL serological pipette (Thermo Fisher Scientific, catalog number: 170367N)
5. Cell scraper (Corning, catalog number: 3010)
6. High-binding ELISA plate (JET-Bio, catalog number: FEP100096)
7. 1.5 mL microfuge tube (Axygen, catalog number: MCT-150-C)
8. 2.0 mL dolphin microcentrifuge tube (Sigma-Aldrich, catalog number: Z717533)
9. 15-well 4%–20% Bis-Tris protein gel (GenScript, catalog number: M42015C)
10. Protein molecular weight standards (Yeasen, catalog number: 20351ES72)

## Equipment

-80 °C freezer (Thermo Fisher Scientific, model: TDE50086FV-ULTS)
-20 °C freezer (Thermo Fisher Scientific, model: ES Series)
3. Biological safety cabinet (Thermo Fisher Scientific, model: 1300 Series A2)
4. Pipette (Rainin, model: LTS Pipette)
5. CO_2_ incubator (Thermo Fisher Scientific, model: Forma Series 3 WJ)
6. Inverted microscope (Nikon, model: Eclipse TS2)
7. Barnstead GenPure Pro ultrapure water system (Thermo Fisher Scientific, model: GenPure UV/UF)
8. Refrigerated centrifuge (Eppendorf, model: 5424R)
9. ThermoMixer (Eppendorf, model: ThermoMixer F1.5)
10. SpeedVac concentrator (Eppendorf, model: Concentrator Plus)
11. pH meter (Mettler Toledo, model: FE20K)
12. Multi-mode microplate reader (BioTek, model: EPOCH)
13. Analytical balance (Mettler Toledo, model: XS64)
14. Vacuum aspirator system (Dragon LAB, model: SAFEVAC)
15. Genie 2 vortex mixer (Scientific Industries, model: G560E)
16. Orbital shaker (Kylin-Bell, model: TS-200)
17. Dry bath incubator (Blue Pard, model: TU-10)
18. Protein electrophoresis system (Bio-Rad, model: PowerPac-Basic)
19. Trans-Blot SD semi-dry electrophoretic transfer cell (Bio-Rad, model: Trans-Blot Turbo)
20. ChemiDoc MP imaging system (Bio-Rad, model: ChemiDoc MP)

## Procedure

**Part I: Metabolic labeling of cellular proteins with alkynyl chemical reporters in living cells**

- **Cell culture**
  1. Seed cells in 12-well plates or 60 mm dishes with normal growth medium (e.g., DMEM supplemented with 10% FBS). Incubate the cells overnight at 37 °C with 5% CO to allow them to adhere and grow.
*Notes:*
    - *Seed the cells to reach 70%–80% confluency by the next day.*
    - *Both HeLa and HEK293T cells were used in this study, but other adherent cell types can also be used.*
    - *Cells in one well of a 12-well plate (approximately 100 μg of protein in cell lysates) are enough for in-gel fluorescence analysis of chemical reporter-labeled proteomes, while cells in a 60 mm dish (approximately 600 μg of protein in cell lysates) are needed for in-gel fluorescence analysis of individual POIs.*
- **Transfection (optional)**
  1. Prepare transfection mixture. For cells cultured in a 12-well plate:
    - In a microcentrifuge tube, dilute 1 μg of plasmid encoding the wild-type (WT) or mutant POI in 100 μL of Opti-MEM.
    - Add 2.5 μL of ViaFect transfection reagent to the diluted DNA.
    - Mix the solution gently by pipetting and incubate the mixture at room temperature for 15 min.
  2. Add the transfection mixture dropwise into the cell media.
  3. Incubate the cells for 6 h at 37 °C in a CO incubator.
*Optional: Transfection is optional; it depends on the experimental requirement. To analyze endogenous proteins labeled by chemical reporters, transfection is not needed. However, transfection to express an exogenous enzyme may be performed to analyze the substrates of that enzyme.*
*Notes:*
    - *The recommended concentration of plasmid stock for transfection is approximately 200–500 ng/μL.*
    - *Gently mix the transfection reagent and plasmid to avoid shear forces that can damage the plasmid DNA.*
    - *Select an appropriate transfection reagent and method based on the cell type.*
- **Metabolic labeling with the chemical reporter**
  1. Carefully replace the existing cell culture media with fresh media, supplemented either with the chemical reporter or the solvent (e.g., DMSO) used to dissolve the chemical reporter as the control.
*Notes:*
    - *In this study, the bioorthogonal chemical reporter alk-16, an alkynyl-functionalized fatty acid analog, was utilized to metabolically label fatty-acylated proteins in living cells.*
    - *Add fresh media gently to avoid dislodging the cells.*
    - *Optimize the concentration of the chemical reporter as needed. For alk-16, a final concentration of 50 µM has been found effective for efficient labeling.*
    - *Choose the appropriate type of cell culture media based on the experiment requirement. In this study, DMEM supplemented with 2% charcoal/dextran-treated fetal bovine serum was used to dissolve alk-16 and incubate the cells. The charcoal/dextran treatment removes lipids from the serum, facilitating the uptake of the fatty acid chemical reporter (e.g., alk-16) without competition from serum lipids.*
    - *Ensure to process a vehicle well without the chemical reporter as the control to evaluate the background fluorescence signal.*
  2. Incubate cells with the chemical reporter for 16 h at 37 °C.
*Notes:*
    - *The incubation time can be optimized and adjusted based on specific experimental needs.*
    - *For different samples, ensure that the labeling period and incubation temperature are consistent.*
  3. After the incubation, discard the medium and resuspend the cells in 1 mL of ice-cold PBS by gently pipetting up and down. Centrifuge at 4,000× *g* for 2 min at 4 °C and discard the supernatant. Add fresh ice-cold PBS, resuspend the cells again by pipetting, and centrifuge once more. Remove the supernatant and collect the cell pellet.
*Notes:*
    - *Use a cell scraper to detach adherent cells, avoiding trypsin as it may degrade cell surface proteins.*
    - *Ensure thorough washing to remove any residual media and chemical reporters (e.g., alk-16).*
    - *Handle the cells gently to avoid cell damage.*
  4. If not proceeding immediately to the following experiments, freeze the cell pellets in liquid nitrogen and store them at -80°C until further use.

**Part II: In-gel fluorescence analysis of chemical reporter-labeled proteomes**

- **Cell lysis and protein quantification**
  1. Lyse the cells labeled with chemical reporters by adding 50 μL of 1% SDS lysis buffer (see Recipes), supplemented with EDTA-free protease inhibitor cocktail and 0.2 μL of benzonase (50 U).
*Notes:*
    - *Benzonase degrades nucleic acids to reduce viscosity.*
    - *Ensure that the lysis buffer is freshly prepared and supplemented with the protease inhibitor cocktail immediately before use. Avoid using protease inhibitors containing EDTA, as EDTA is incompatible with the click reaction.*
    - *It is preferable to use the lysis buffer in a volume to achieve a protein concentration above 2 mg/mL in the cell lysate.*
  2. Vortex vigorously to ensure complete lysis.
  3. Centrifuge the lysates at 12,000× *g* for 20 min at room temperature. Collect the supernatant and discard the pellet (cell debris).
  4. Determine the protein concentration using a standard BCA assay.
*Note: Quantification of protein concentration is crucial for subsequent steps.*
  5. Aliquot equal amounts of protein (e.g., 100 μg) into 1.5 mL tubes and adjust the volume to 50 μL with the 1% SDS lysis buffer.
  6. Add 39 μL of HEPES buffer (see Recipes) to bring the total volume to 89 μL.
- **Click reaction**
  1. Prepare the click reaction cocktail by adding the following components sequentially into an Eppendorf tube: 10 μL of 5 mM azido-rhodamine in DMSO, 25 μL of 2 mM TBTA in DMSO/t-BuOH, 10 μL of 50 mM TCEP in H_2_O, and 10 μL of 50 mM CuSO_4_·5H_2_O in H_2_O. Vortex the mixture thoroughly.
*Notes:*
    - Prepare TCEP and CuSO_4_·5H_2_O solutions freshly.
    - Azido-rhodamine should be protected from light.
  2. Add 11 μL of the click reaction cocktail to each lysate sample prepared in step A of part II. Vortex to mix thoroughly.
  3. Incubate the reaction mixture in the dark at room temperature for 2 h.
*Note: A 2-h incubation time ensures the click reaction is as thorough as possible; however, the duration can be adjusted based on specific experimental needs, such as reducing it to 1 h.*
- **Protein precipitation and washing**
  1. Terminate the click reaction by adding 500 μL of ice-cold methanol. Store the mixture at -20 °C overnight.
*Note: Methanol should be ice-cold to ensure efficient protein precipitation.*
  2. Centrifuge the mixture at 20,000× *g* for 20 min at 4 °C.
  3. Discard the supernatant carefully and retain the protein pellet.
*Note: Remove the supernatant cautiously to avoid disturbing the protein pellet.*
  4. Wash the protein pellets twice by adding 1 mL of ice-cold methanol, gently inverting the tube, and centrifuging at 20,000× *g* for 20 min at 4 °C. The protein pellet should be at the bottom of the tube.
  5. Air-dry the protein pellets on the bench for 60 min at room temperature.
*Note: Ensure that pellets are completely dry before proceeding to subsequent steps.*
- **Sample preparation for SDS-PAGE**
  1. Resuspend the protein pellet in 57.5 μL of 4% SDS buffer (see Recipes). Shake the samples for 15 min at 1,500 rpm to ensure complete dissolving.
*Notes:*
    - *Ensure complete dissolution of the protein pellet for subsequent analysis.*
    - *Use a bath sonicator to aid solubilization if necessary. Sonication helps to dissolve the protein pellets.*
  2. Add 12.5 μL of 2 M NH_2_OH to each sample.
*Optional: NH_2_OH selectively cleaves thioester bonds but not amide bonds, so treatment with NH_2_OH can minimize interference from S-palmitoylated proteins. If NH_2_OH treatment is not needed, add 70 μL of 4% SDS buffer (see Recipes) directly to dissolve the protein pellet in the previous step.*
  3. Add 25 μL of 4× SDS-PAGE loading buffer (see Recipes) and 5 μL of bond-breaker TCEP solution.
  4. Heat the samples at 95 °C for 5 min to denature the proteins. Briefly vortex and then centrifuge the samples for 1 min at 10,000× *g* at room temperature.
- **SDS-PAGE and in-gel fluorescence visualization**
  1. Load 20 μg of protein per lane onto a 4%–20% Bis-Tris gel. Run the gel for 1 h at 140 V.
*Note: Use a fluorescent protein ladder as the molecular weight standard to facilitate accurate size determination during fluorescence scanning.*
  2. Destain the gel by rocking it for 1 h in destaining buffer (see Recipes) at room temperature.
*Note: The destaining step removes the remaining traces of the loading buffer and unreacted rhodamine.*
  3. Scan the gel on a ChemiDoc MP imager using “rhodamine” mode.
  4. Following fluorescence scanning, stain the gel with Coomassie Brilliant Blue staining to confirm equal protein loading of samples.
*Notes:*
    - *Coomassie Brilliant Blue staining provides a visual check for total protein amount and loading consistency.*
    - *As an example, the result for in-gel fluorescence analysis of fatty-acylated proteins using metabolic labeling with the bioorthogonal chemical reporter alk-16 and click reaction is shown in Figure 2A.*

**Part III: Metabolic labeling of the protein of interest with alkynyl chemical reporters in living cells**

- **Cell culture**
  1. Seed cells in 60 mm dishes with normal growth medium as described in step A of part I.
- **Transfection**
  1. Prepare transfection mixture. For cells cultured in a 60 mm dish:
    - In a microcentrifuge tube, dilute a total of 4 μg of plasmid encoding the WT or mutant POI in 400 μL of Opti-MEM.
    - Add 10 μL of ViaFect transfection reagent to the diluted DNA.
    - Mix the solution gently by pipetting and incubate the mixture at room temperature for 15 min.
*Note: In this study, HEK293T cells were transfected with HA-tagged Ras-related protein R-Ras (RRAS) to validate the fatty-acylation of RRAS by in-gel fluorescence assay.*
  2. Add the transfection mixture dropwise into the cell media.
  3. Incubate the cells for 6 h at 37 °C in a CO incubator.
*Notes:*
    - *The transfection procedure is essential for overexpressing the POI with an affinity tag. In this study, HEK293T cells were transfected with HA-tagged RRAS to validate the fatty-acylation of RRAS by in-gel fluorescence assay.*
    - *In initial experiments, it is crucial to check transfection efficiency and ensure the effective expression of the POI. If the POI is fused with a fluorescent protein tag, such as GFP or RFP, use fluorescence microscopy to assess the transfection efficiency and expression level of the POI. If the POI does not have a fluorescent tag, perform western blotting to evaluate the protein expression level.*
- **Metabolic labeling with the chemical reporter**
  1. Metabolic labeling of proteins with chemical reporters in living cells and harvest cells as described in Step C of part I.

**Part IV: In-gel fluorescence analysis of the protein of interest**

- **Cell lysis and protein quantification**
  1. Lyse the cells labeled with chemical reporters by adding 200 μL of RIPA buffer (see Recipes) supplemented with EDTA-free protease inhibitor cocktail and 0.4 μL of benzonase (100 U). Vortex vigorously and then rotate at 4 °C for 30 min.
*Note: Other lysis buffers compatible with immunoprecipitation may be used in place of RIPA buffer.*
  2. Centrifuge the lysates at 12,000× *g* for 20 min at 4 °C. Collect the supernatant and discard the pellet (cell debris).
  3. Quantify protein concentration using a standard BCA assay.
  4. Aliquot equal amounts of protein (e.g., 600 μg) into 2.0 mL dolphin microcentrifuge tubes and adjust the volume with the RIPA buffer to 1.5 mg/mL.
- **Immunoprecipitation**
  1. For the immunoprecipitation of proteins tagged with HA/GFP/FLAG, use the corresponding anti-HA/GFP/FLAG agarose beads. Equilibrate the beads by washing them with PBS before use.
  2. Add the agarose beads (e.g., 20 μL of bead slurry per 600 μg of cell lysate) to the lysate and incubate the mixture on a rotator at 4 °C for 2 h. Gentle rotation is essential for efficient binding of the tagged protein to the beads.
  3. After incubation, centrifuge the beads–lysate mixture at 8,000× *g* for 1 min at 4 °C to pellet the beads. Carefully remove the supernatant without disturbing the bead pellet.
  4. Wash the beads three times with 1 mL of chilled RIPA buffer to remove non-specifically bound proteins. For each wash, gently rotate the beads on a rotator at 4 °C for 5 min, then centrifuge at 8,000× *g* for 1 min at 4 °C.
  5. After washing with RIPA buffer, wash the beads three additional times with 1 mL of chilled PBS.
  6. Resuspend the beads in 44.5 μL of PBS.
- **Click reaction**
  1. Add 5.5 μL of freshly prepared click reaction cocktail (as described in step B1 of part II) to the resuspended beads.
  2. Incubate the reaction mixture in the dark for 2 h at room temperature.
  3. Wash the agarose beads three times with 1 mL of RIPA buffer.
- **Sample preparation for SDS-PAGE**
  1. Resuspend the beads with 17.25 μL of ultrapure water, 7.5 μL of 4× SDS-PAGE loading buffer, and 1.5 μL of bond-breaker TCEP solution.
  2. Add 3.75 μL of 2 M NH_2_OH to each sample.
*Optional: If NH_2_OH treatment is not required, add 21 μL of ultrapure water directly in the previous step.*
  3. Heat the samples at 95 °C for 5 min to denature the proteins. Briefly vortex and then centrifuge the samples for 1 min at 8,000× *g* at room temperature to pellet agarose beads.
- **SDS-PAGE and in-gel fluorescence visualization**
  1. Load 20 μL of the supernatant per lane onto a 4%–20% Bis-Tris gel for SDS-PAGE and run at 140 V for 1 h. This gel is used for fluorescent gel scanning.
  2. Add 20 μL of 1× SDS-PAGE loading buffer to the remaining sample, vortex, and centrifuge the samples at 8,000× *g* for 1 min at room temperature. Load 20 μL of the supernatant per lane onto another 4%–20% Bis-Tris gel for SDS-PAGE and run at 140 V for 1 h. This gel serves as a control to confirm protein loading through western blotting analysis.
  3. After running, destain the first gel and perform fluorescence scanning and Coomassie Brilliant Blue staining as described in steps E2–4 of part II.
  4. Transfer the proteins from the second gel onto a nitrocellulose membrane and detect the POI using standard western blotting procedures.
*Note: As an example, the result for in-gel fluorescence analysis of fatty-acylation of HA-tagged RRAS is shown in Figure 2B.*
    **Figure 2.**
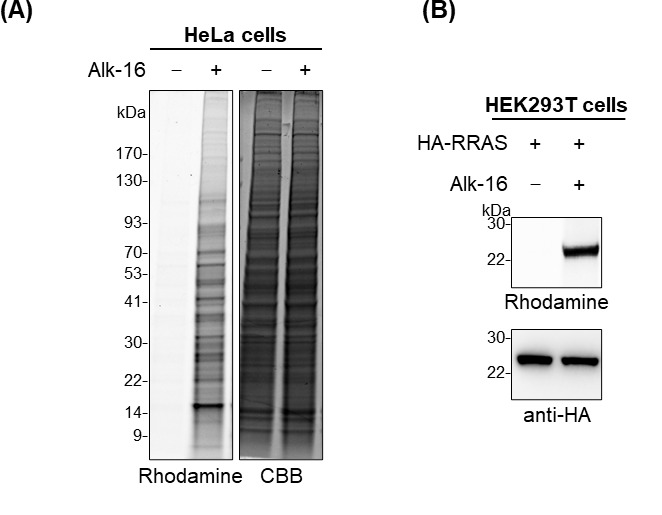
Results for in-gel fluorescence analysis of alk-16-labeled fatty-acylated proteins. (A) In-gel fluorescence analysis of alk-16-labeled fatty-acylated proteins in HeLa cells. Coomassie Brilliant Blue (CBB) staining is shown to confirm equal protein loading. (B) Validation of fatty-acylation of Ras-related protein R-Ras (RRAS) by in-gel fluorescence analysis. HEK293T cells were transfected with HA-tagged RRAS. The cells were metabolically labeled with alk-16 and subjected to immunoprecipitation and in-gel fluorescence assay. Anti-HA immunoblotting is shown to confirm sample loading.

## Validation of protocol

This protocol or parts of it has been used and validated in the following research article:

- Xu et al. [7]. Chemical Proteomics Reveals N^ε^-Fatty-Acylation of Septins by Rho Inactivation Domain (RID) of the Vibrio MARTX Toxin to Alter Septin Localization and Organization. Mol Cell Proteomics.
  Rho inactivation domain (RID) from *Vibrio cholerae* and *Vibrio vulnificus* efficiently catalyzes the covalent attachment of long-chain fatty acyl groups (e.g., palmitoyl, stearyl) to the ε-amino group of lysine residues on substrate proteins in host cells [7, 10] (Figure 3A). To globally profile RID-mediated *N* ^ε^-fatty acylation, HeLa cells were transfected with either wild-type RID (RID-WT) or the inactive mutant RID-C2835A (RID-CA), incubated with alk-16 overnight, and then lysed. Proteins in the lysates were subjected to click chemistry with azido-rhodamine for in-gel fluorescence analysis, as described in parts I and II (Figure 3B). To reduce interference from *S*-palmitoylated proteins, lysates were treated with NH_2_OH to selectively cleave thioester bonds. The in-gel fluorescence revealed specific alk-16 labeling of many proteins in RID-WT lysates, but not in RID-CA lysates (Figure 3C), indicating that RID mediates *N* ^ε^-fatty-acylation of these host proteins in living cells.
  To further validate that RID mediates the *N* ^ε^-fatty-acylation of Ras-related protein Rac1 (RAC1), an in-gel fluorescence assay was performed. Specifically, HEK293T cells were co-transfected to express RID and HA-tagged RAC1, followed by labeling with alk-16. Cell lysates were subjected to immunoprecipitation, click-reacted with azido-rhodamine, and analyzed by in-gel fluorescence after NH_2_OH treatment, as described in parts III and IV (Figure 4A). The results showed that RAC1 was labeled by Alk-16 only in the presence of RID-WT, but not upon expression of RID-CA (Figure 4B), confirming that RID catalyzes the *N* ^ε^-fatty acylation of RAC1.
  **Figure 3.**
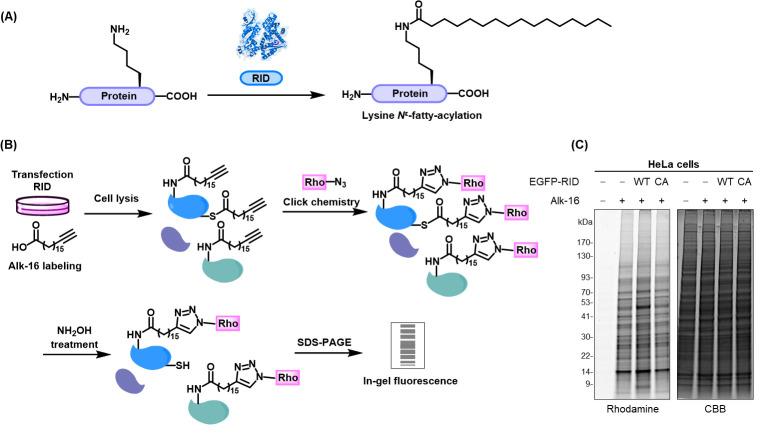
Workflow and result for in-gel fluorescence analysis of alk-16-labeled fatty-acylated proteins. (A) RID of the *Vibrio* MARTX toxin catalyzes *N* ^ε^-fatty-acylation modifications on lysines of its substrate proteins. (B) Schematic for in-gel fluorescence analysis of RID-mediated *N* ^ε^-fatty-acylation of proteins using metabolic labeling with the bioorthogonal chemical reporter alk-16 and click reaction. (C) In-gel fluorescence analysis of alk-16-labeled fatty-acylated proteins in mock- and RID (WT or CA)-transfected HeLa cells. Coomassie Brilliant Blue (CBB) staining is shown to confirm equal protein loading.
  **Figure 4.**
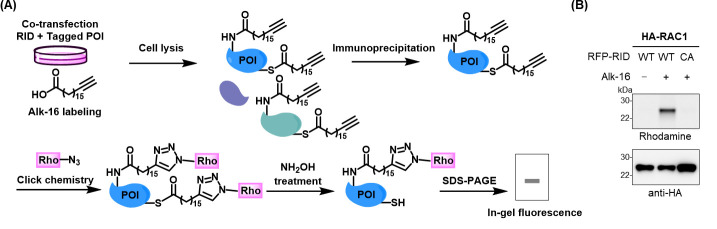
Workflow and result for in-gel fluorescence analysis of RID-mediated *N* ^ε^-fatty-acylation of HA-tagged RAC1. (A) Workflow for analysis of RID-mediated *N* ^ε^-fatty-acylation of candidate proteins of interest (POI). HEK293T cells were co-transfected with RID and individual tagged POI, metabolically labeled with alk-16, and lysed. The cell lysates were subjected to immunoprecipitation, click reaction with azido-rhodamine, NH_2_OH treatment to cleave thioester bonds, and in-gel fluorescence analysis. (B) Validation of RID-mediated *N* ^ε^-fatty-acylation of RAC1 by in-gel fluorescence analysis. Samples were prepared as in (A). Anti-HA immunoblotting is shown to confirm sample loading.

## General notes and troubleshooting

**Troubleshooting**

- **Cells unhealthy after transfection (step B of parts I and III)**
  1. Ensure cell confluency is around 70% before transfection.
  2. Adjust the transfection reagent-to-plasmid ratio and consider shortening the transfection time.
  3. Replace the media 6 h after transfection.
  4. Check if overexpression of the POI is toxic to the cells.
- **Cells detaching during media change (step C1 of parts I and III)**
  1. Gently aspirate and replace the media, avoiding direct pipetting onto the cells.
  2. Slowly add media near the edge of the dish to minimize disruption to the cell monolayer.
  3. Consider using an electronic pipette aid with a gravity drain feature.
  4. Using poly-D-lysine-coated culture dishes may improve cell attachment.
- **Click chemistry reaction not proceeding efficiently (step B of part II and step C of part IV)**
  1. Always use freshly prepared TCEP and CuSO_4_ solutions, as TCEP may oxidize over time and Cu(I) is critical for the reaction. Prepare these solutions just before use to ensure optimal reaction efficiency.
- **Poor protein precipitation after the click reaction (step C of part II)**
  1. Ensure that methanol is ice-cold and has been stored at -20 °C overnight before centrifugation.
  2. Increase methanol volume if needed to improve precipitation.
- **Incomplete resolubilization of the protein pellet (step D1 of part II)**
  1. Ensure the protein pellet is completely dry before dissolving.
  2. Use 4% SDS buffer combined with sonication to fully resolubilize the protein.
- **High background fluorescence in fluorescence gel scans (step E3 of parts II and IV)**
  1. Wash the protein pellet twice with ice-cold methanol during the precipitation step to remove unreacted azido-rhodamine.
  2. Thoroughly destain the gel after SDS-PAGE to eliminate residual fluorescence.
  3. Handle the gel by its edges with clean gloves to avoid smudges or fingerprints.
- **Failure to detect the protein of interest in immunoprecipitation (step B of part IV)**
  1. If low protein expression levels are suspected or epitope loss occurs during lysis, verify protein expression on a small sample before immunoprecipitation.
  2. If the protein is localized in the nucleus or detergent-resistant membranes (DRMs), using 1% SDS lysis buffer may be necessary to lyse the cells. Dilute SDS to 0.1% before incubating with beads.
  3. For inefficient binding, extend the incubation time and increase the amount of beads used.
  4. If protein loss occurs during washing, use gentler washing conditions and avoid vigorous vortexing of the beads.
